# Trends of Ischemic Heart Disease and Cerebrovascular Disease in Active Component Female Service Members, 2014–2023

**Published:** 2024-11-20

**Authors:** Valentina Donici, Shauna L. Stahlman, Michael T. Fan, Richard S. Langton

**Affiliations:** 1 Uniformed University of the Health Sciences, Bethesda, MD; 2 Epidemiology and Analysis Branch, Armed Forces Health Surveillance Division, Defense Health Agency, Silver Spring, MD; 3 Armed Forces Health Surveillance Division, Defense Health Agency, U.S. Department of Defense

## Abstract

This study summarizes trends from 2014 through 2023 in the incidence of ischemic heart disease and cerebrovascular heart disease among U.S. active component female service members and identifies potential military-specific risk factors for these conditions. Female-specific risk factors, such as mental health, for ischemic heart and cerebrovascular diseases have only recently been recognized. Crude incidence rates were assessed for each outcome and Poisson regression was used to calculate adjusted incidence rates, controlling for multiple covariates. After adjustment, non-Hispanic Black active component U.S. service women had higher rates for both ischemic heart disease and cerebrovascular disease compared to non-Hispanic White service women (IRR=1.68 and 1.24, respectively). A prior diagnosis of depressive or anxiety disorder resulted in a 90% increased rate of ischemic heart disease and 70% increased rate of cerebrovascular disease. Air Force members had a 55% increased rate of ischemic heart disease. This study identifies both military-specific and demographic risk factors for these 2 cardiovascular diseases and demonstrates potential opportunity for early age preventive care, even among a relatively young and healthy population.

**What are the new findings?:**

Among active component U.S. service women, incidence of ischemic heart disease increased between 2014 (31.2 per 100,000 person-years) and 2019 (54.7 per 100,000 p-yrs), while incidence of cerebrovascular disease decreased during that period and increased between 2019 (28.5 per 100,000 p-yrs) and 2023 (46.4 per 100,000 p-yrs). Older age, non-Hispanic Black race and ethnicity, and prior depressive or anxiety disorder diagnosis were identified as potential risk factors for both outcomes.

**What is the impact on readiness and force health protection?:**

Cardiovascular diseases are often overlooked among women, but this study identified both military-specific (e.g., branch of service, prior depressive disorder diagnosis) as well as demographic (e.g., race, age) potential risk factors, and demonstrated there may be an opportunity for preventive intervention even among this relatively young and healthy population.

## 1 BACKGROUND

A recent report on coronary heart disease in the U.S. noted that in 2018 the prevalence of ischemic heart disease in women was 4.7%.^1^ While overall mortality from ischemic heart disease in the U.S. improved from 1979 to 2011 with medical advances, it has, unfortunately, stagnated for women under 55 years of age.^2^ Studies have advocated for a closer analysis of the impact of new “emerging nontraditional” atherosclerotic cardiovascular disease risk factors, 1 of which is mental health disorder and psychological trauma.^3^ Ebrahimi noted in 2017 that rates of cardiac disease mortality among female veterans were higher by 26.4% than among civilian women.^4^ The authors of that study hypothesized that the causes are likely multifactorial and include treatment non-adherence, higher prevalence of cardiovascular risk factors, and greater clinical complexity within the veteran female population.

Cerebrovascular disease is also an important source of morbidity and mortality among women, with many etiologic and pathologic pathways that are not optimally understood. It is reported that 54.2% of the 7 million stroke survivors in the U.S. are women.^5^ Consequently, it is important to study the risk factors that may disproportionately affect women.

The U.S. Department of Defense has a growing female military population that functions in a variety of military settings, including deployments, combat trades, and other military-specific activities. These activities may increase individuals’ chronic stress and risk for post-traumatic stress disorder (PTSD), as noted by Bourassa and by Cohen, in their respective studies on 9/11 and military veterans.^6,7^ Identifying potential correlations between these military experiences and rates of cerebrovascular disease could identify new areas of focus, to mitigate the effects of these risk factors on the long-term health of female service members. Given the paucity of existing data, this study aims to fill a gap in the current knowledge of active duty women’s health.

The primary objective of this study was to identify trends in incidence of ischemic heart disease and cerebrovascular disease among U.S. active component female service members between 2014 and 2023. The secondary objective was to identify potential military-specific risk factors for these conditions among U.S. active component female service members.

## 2 METHODS

This surveillance study examined a retrospective cohort of active component female service members in the U.S. Army, Air Force, Navy, Marine Corps, and Air Force (including Space Force) between January 1, 2014 and December 31, 2023. The data source was the Defense Medical Surveillance System (DMSS).

The outcomes assessed were ischemic heart disease and cerebrovascular disease (**Table 1**). To qualify as a case, an individual had to have an inpatient record with a diagnosis in the first or second diagnostic position, or at least 2 outpatient visits within a 60-day range with a diagnosis in the first or second diagnostic position, with a qualifying International Classification of Diseases, 9th Revision, Clinical Module (ICD-9-CM) or 10th Revision (ICD-10-CM) diagnosis. Incidence was calculated per 100,000 person-years (p-yrs) of active component service. Person-years were included from the time a woman enrolled in active component service until the time she separated from service, or the end of the surveillance period on December 31, 2023, whichever occurred first. In addition, prevalent cases (i.e., incident cases prior to January 1, 2014) were excluded separately for each outcome, and person-time was censored at the incident diagnosis date.

The risk factors assessed included history of diagnosis of hyperlipidemia, hypertension, diabetes, obesity, tobacco use or nicotine dependence, depression, anxiety, PTSD, and sleep apnea (**Table 1**). An individual was defined as having a history of diagnosis for each of these conditions if the individual had at least 1 inpatient or 1 outpatient encounter with a specified diagnosis in any diagnostic position, including any diagnoses since the individual joined military service. Additional demographic covariates included age, race and ethnicity, service branch, rank, military occupation, and deployment history. The covariates were chosen based on known traditional cardiovascular factors, along with military-specific risk factors identified by the authors.

Crude (i.e., unadjusted) incidence rates were calculated per 100,000 p-yrs. A multivariable Poisson regression model was used to calculate adjusted incidence rate ratios, separately, for the outcomes of ischemic heart disease and cerebrovascular disease. Age, race and ethnicity, service branch, military occupation, rank, deployment history, history of a prior risk factor (e.g., hyperlipidemia, hypertension, diabetes, obesity, tobacco use or nicotine dependence) diagnosis, history of anxiety or depression diagnosis, and history of PTSD diagnosis were included as independent variables in the model. Reference categories were selected based on the largest number of individuals for a given category. All analyses were performed using SAS Enterprise Guide version 8.4.

## 3 RESULTS

**Study Population**

The population characteristics of U.S. active component female service members are described in prior *MSMR* reports.^18^ This study included a total of 2,154,313.5 female active component p-yrs from 2014 to 2023, with 65.3% of the study population under 30 years of age and 26.5% from 30 to 39 years of age. Less than half (42.2%) were non-Hispanic White, 24.8% were non-Hispanic Black, and 18.8% were Hispanic.

**Ischemic Heart Disease**

**Figure 1:**
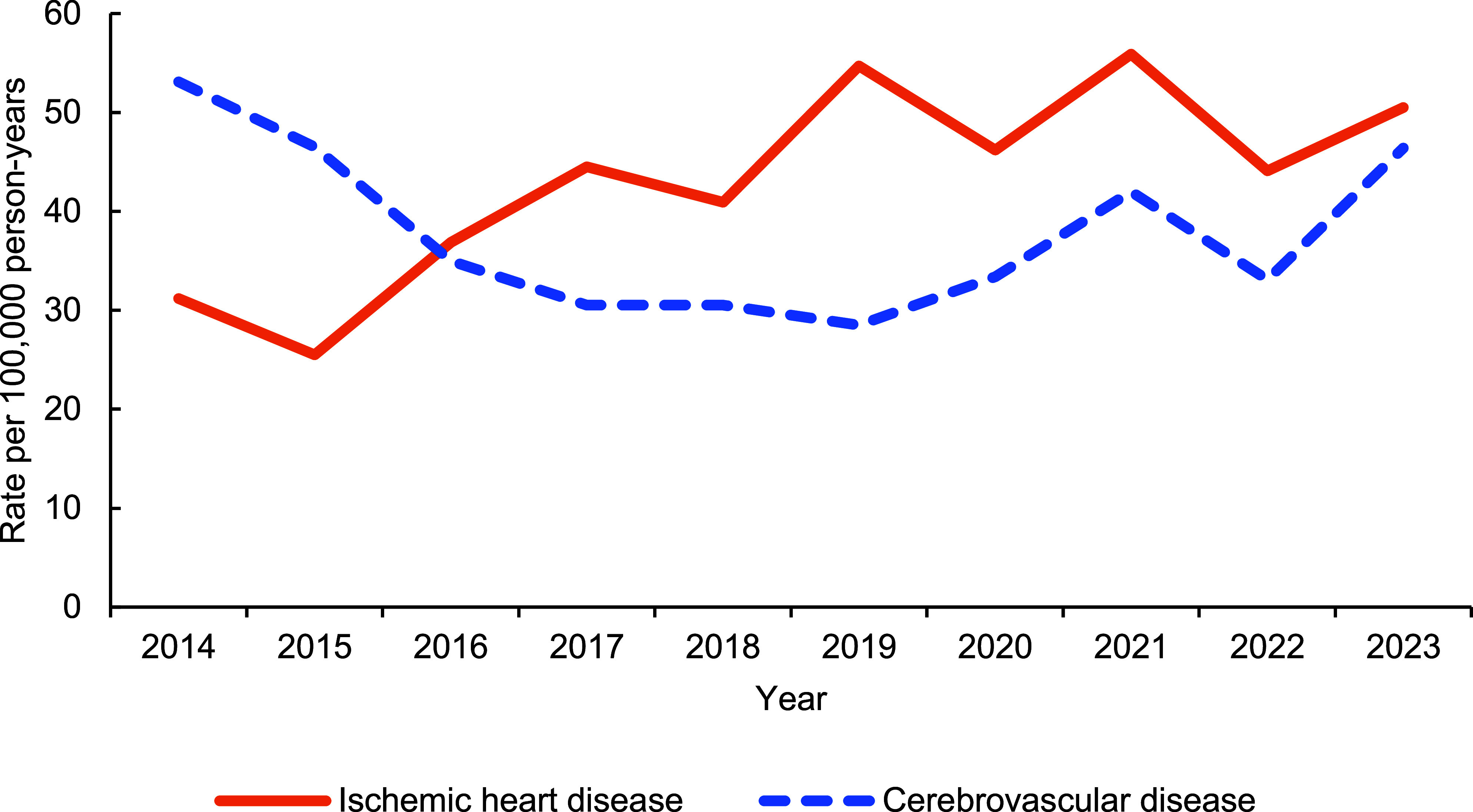
Incidence of Ischemic Heart Disease and Cerebrovascular Disease, Active Component Service Women, 2014–2023

A total of 936 incident cases of ischemic heart disease were identified during the surveillance period (**Table 2**), resulting in a rate of 43.4 cases per 100,000 p-yrs. Over the observed 10 years, the total annual rate increased between 2014 and 2018, then stabilized between 2018 and 2023 (**Figure 1**).

The rate of ischemic heart disease of the 30-39-year age group was twice as high as the rate of the under-30 age group (48.3 and 23.5 cases per 100,000 p-yrs, respectively). Non-Hispanic Black service women had 1.7 times the rate of ischemic heart disease compared to non-Hispanic White service women. Air Force and Space Force members had the highest rates compared to other branches, and those in health care occupations had a higher rate compared to other military occupations. Senior enlisted members and senior officers had higher rates than junior enlisted members or junior officers. Those with a prior diagnosis of a depressive or anxiety disorder or PTSD had 3.1, 2.5, and 1.9 times, respectively, the rate of ischemic heart disease compared to those without such diagnoses.

After adjusting for potential confounders, non-Hispanic Black women had a 68% higher rate compared to non-Hispanic White women (**Table 3**). In addition, prior diagnosis of a depressive or anxiety disorder resulted in a 90% increased rate of ischemic heart disease. Deployment history, military occupation, and prior PTSD diagnosis were not, however, significantly associated with ischemic heart disease after adjustment for other factors. Compared to junior enlisted personnel, junior officers had a 27% smaller rate, while senior officers were not statistically significantly different from junior enlisted service members.

**Cerebrovascular Disease**

There were 814 cases of cerebrovascular disease during the surveillance period (37.8 cases per 100,000 p-yrs.). The overall annual rate had a significant dip between 2016 and 2020, but then progressively returned to the 2014 rate thereafter (**Figure 1**). The rate for the 30-39-year age group was more than double of the under-30-year age group (48.8 and 23.8 cases per 100,000 p-yrs, respectively). Compared to their respective counterparts, unadjusted rates were highest among non-Hispanic Black female service members, senior officers, health care workers, and those with multiple prior deployments.

After adjustment, non-Hispanic Black service women continued to have a higher rate of cerebrovascular disease when compared to non-Hispanic White service women (1.24 aIRR), and those with a prior diagnosis of PTSD, depression, or anxiety continued to have a significantly increased rate of cerebrovascular disease compared to those without a history of diagnosis for those conditions. Those in pilot or air crews and health care occupations had a non-statistically significant increased rate of cerebrovascular disease (24% and 19%, respectively) when compared to communications and intelligence occupations. Although the crude rate for senior officers was more than double of that of junior enlisted service members, after adjustment senior officers had a 40% lesser rate than junior enlisted members.

## 4 DISCUSSION

**Ischemic Heart Disease**

This study found that non-Hispanic Black women and those aged 30 years and older had higher adjusted rates of ischemic heart disease among active component service women. The finding of higher rates among non-Hispanic Black women is consistent with studies published for the U.S. population.^8^ It was also noted that the rate increased by 48% in the 30-39-year age range when compared to the under-30 age group, which was similar to findings from the Veterans Administration study by Chen that that found increased cardiovascular disease risk starting as early as age 30 years.^9^ This is important, as it suggests there is opportunity for intervention at younger ages to prevent risk of developing cardiovascular disease in later life.

The Air Force’s increased incidence rate of ischemic heart disease correlates with findings from literature on airline pilots and cockpit crews.^10^ Those populations are prone to cardiovascular disease due to prolonged sedentary posture, occupational stress and emotional tension, forced operational speed, acceleration, frequent time zone changes, and noise and unbalanced diets.^10^ The Marine Corps, on the other hand, had a significantly lower rate than other services, which may be due to reduced symptom reporting by patients to health services, as well as a ‘healthy-ier warrior’ effect, since the Marine Corps has stringent fitness requirements. Findings from this study also suggest a correlation between socioeconomic status (i.e., pay scale) and risk for heart diseases, because junior enlisted members had higher adjusted rates of both outcomes compared to junior officers.

This study also found that a prior diagnosis of depression or anxiety almost doubled risk of ischemic heart disease among active component service women. Multiple studies have pointed to the link between depression and coronary artery disease, with a stronger association observed in younger women.^3,17^ Prior diagnosis of PTSD did not show a statistically significant association with ischemic heart disease. It is possible, however, that the association between PTSD and ischemic heart disease was diminished by adjusting for depressive disorder diagnosis, which could be correlated with PTSD.^11^

**Cerebrovascular Disease**

Consistent with other studies,^12^ non-Hispanic Black women have increased adjusted rates for cerebrovascular disease when compared to non-Hispanic White women. The junior and senior officer groups have lower adjusted rates when compared to junior enlisted members, which, once again, may indicate a root cause stemming from social determinants of health such as financial stability or lower levels of education. A prior diagnosis of depression, anxiety, or PTSD all present an elevated incidence rate of cardiovascular disease, in accordance with the National Institutes of Health’s statement that anxiety, depression, and high stress levels may raise risk of stroke.^13^

Although higher unadjusted rates of ischemic heart disease and cerebrovascular disease were observed among those with multiple deployments, after adjusting for potential confounders this was not the case, which suggests that the crude association may have been confounded by age or other covariates such as depression.

Most of the limitations of this study are due to the use of ICD code diagnoses as the only source of information on the presence of a risk factor or an outcome. Obesity and smoking are likely under-represented, as risk factor cases are not identified if not documented as a concern during a patient encounter. The addition of periodic health assessment data would likely result in additional identification of risk factor cases, but these data prior to 2018 are not available in DMSS. Outcomes may be underestimated due to the use of surveillance case definitions that require 2 outpatient encounters within 60 days. Also, female-specific risk factors such as reproductive health (e.g., contraception or pregnancy) were not analyzed. Finally, DMSS lacks Asian/Pacific Islander data for the Air Force, which forced the inclusion of this population in the ‘Other’ race and ethnicity category.

The risk factors for both ischemic heart disease and cerebrovascular disease are complex and tightly intertwined. A recommendation would be to investigate more thoroughly the effect of each separate mental health diagnosis (e.g., depression, anxiety) with further exploration into the potential association with deployment history, operational PTSD, and development of cardiovascular disease later in life. Cardiovascular diseases are often overlooked among women, but this study identified both military-specific (e.g., service branch, prior depressive disorder diagnosis) and demographic (e.g., race, age) potential risk factors, demonstrating future opportunity for preventive intervention among even this relatively young and healthy population.

## Figures and Tables

**Table 1:** ICD-10-CM Codes Utilized to Identify Outcome and Exposure Variables

	ICD-10-CM	ICD-9-CM
Outcome		
Ischemic heart disease	I20.*–I25.*	410.*–414.*
Cerebrovascular disease	I60.*–I66.*, I67.2	430.*–436.*, 437.0, 437.1, 437.2
Exposures		
Essential hypertension	I10.0, I16.*	401.*
Hyperlipidemia	E78.0*–E78.5*	272.0–272.4
Obesity	E10.*, E11.*, R73.*	250.*, 790.2*
Diabetes mellitus or abnormal glucose level	E10.*, E11.*, R73.*	250.*, 790.2*
Depressive disorder	F32.*, F33.*, F34.0, F34.1, F34.8, F34.9, F39.0, F34.81, F34.89	296.2, 296.21, 296.22, 296.23, 296.24, 296.25, 296.26, 296.20, 311.0, 296.3, 296.30, 296.31, 296.32, 296.33, 296.35, 296.36, 296.99, 300.4, 296.90, 296.9
Anxiety disorder	F40.*, F41.*, F42.*	300.22, 300.21, 300.23, 300.29, 300.20, 300.01, 300.02, 300.09, 300.00, 300.3
PTSD	F43.1, F43.10, F43.11, F43.12	309.81
Smoking or nicotine dependence	F17.2*, Z72.0, Z87.891	305.1, V15.82
Sleep apnea	G47.3*	780.51, 780.57, 327.2*, 780.53

Abbreviations: ICD-10-COM, International Classification of Diseases, 10th edition, Clinical Modification; ICD-9-COM, International Classification of Diseases, 9th edition, Clinical Modification; PTSD, post-traumatic stress disorder.

* Includes all subsequent digits and characters.

**Table 2:** Incident Counts and Rates of Cardiovascular Disease by Type and Demographic and Military Characteristics, Active Component, U.S. Armed Forces, 2014–2023

	Ischemic Heart Disease		Cerebrovascular Disease	
	No.	Rate^a^	No.	Rate^a^
Total	936	43.4	814	37.8
Inpatient	193	9.0	231	10.7
Outpatient	743	34.5	583	27.1
Race and ethnicity				
White, non-Hispanic	340	37.4	332	36.5
Black, non-Hispanic	342	64.0	272	50.9
Hispanic	139	34.3	112	27.6
Other/unknown	115	37.6	98	32.1
Age group, y				
<30	331	23.5	335	23.8
30–39	276	48.3	279	48.8
40–49	227	146.2	151	97.3
50+	102	471.5	49	224.3
Service branch				
Army	341	48.2	301	42.6
Navy	180	28.1	219	34.2
Air Force/Space Force	394	60.6	257	39.5
Marine Corps	21	13.5	37	23.8
Rank				
Junior enlisted (E1-E4)	248	25.8	257	26.8
Senior enlisted (E4-E9)	431	55.7	395	51.1
Junior officer (O1-O3)	73	26.9	57	21.0
Senior officer (O4-O10)	168	127.0	92	69.6
Warrant officer (W01-W05)	16	93.5	13	76.2
Occupation				
Combat-specific	14	24.9	12	21.4
Armor/motor transport	21	31.2	18	26.8
Pilot/air crew	13	39.0	9	27.0
Repair/engineering	141	32.9	145	33.8
Communications/intelligence	310	45.0	277	40.3
Health care	241	59.8	205	50.9
Other	196	41.0	148	31.0
Number of prior deployments				
0	449	31.4	434	30.3
1	211	53.7	166	42.3
2+	276	83.6	214	64.8
PTSD history				
Yes	250	73.1	194	56.7
No	686	37.9	620	34.2
Depressive disorder history				
Yes	382	98.4	305	78.6
No	554	31.4	509	28.8
Anxiety disorder history				
Yes	109	102.2	105	98.4
No	827	40.4	709	34.6
Hyperlipidemia				
Yes	272	254.5	192	124.1
No	664	32.4	622	33.4
Hypertension				
Yes	241	230.3	130	124.1
No	695	33.9	684	33.4
Diabetes or abnormal glucose				
Yes	275	103.8	194	73.2
No	661	35.0	620	32.8
Obesity				
Yes	145	182.9	98	123.6
No	791	38.1	716	34.5
Tobacco use or nicotine dependance				
Yes	319	91.1	279	79.8
No	617	34.2	535	29.7
Obstructive sleep apnea				
Yes	174	183.0	119	125.1
No	762	37.0	695	33.8

Abbreviations: No., number; y, year; PTSD, post-traumatic stress disorder; p-yrs, person-years.

^a^Rate per 100,000 p-yrs.

**Table 3:** Adjusted Incidence Rate Ratios for Cardiovascular Disease, Active Component Service Women, U.S. Armed Forces, 2014–2023

	Ischemic Heart Disease			Cerebrovascular Disease		
	aIRR	95% LL	95% UL	aIRR	95% LL	95% UL
Race and ethnicity						
White, non-Hispanic	Reference	–	–	Reference	–	–
Black, non-Hispanic	1.7	1.4	2.0	1.2	1.0	1.5
Hispanic	1.2	1.0	1.5	0.8	0.7	1.0
Other/unknown	1.1	0.9	1.4	0.9	0.7	1.1
Age group, y						
<30	Reference	–	–	Reference	–	–
30–39	1.5	1.2	1.8	1.7	1.4	2.1
40–49	3.8	3.0	4.9	3.0	2.3	3.9
50+	12.0	8.8	16.5	6.9	4.7	10.1
Service branch						
Army	Reference	–	–	Reference	–	–
Navy	0.8	0.7	0.9	1.0	0.8	1.2
Air Force/Space Force	1.6	1.3	1.8	1.0	0.9	1.2
Marine Corps	0.5	0.3	0.8	0.9	0.7	1.3
Rank						
Junior enlisted (E1-E4)	Reference	–	–	Reference	–	–
Senior enlisted (E5-E9)	0.8	0.6	1.0	0.8	0.7	1.0
Junior officer (O1-O3)	0.7	0.6	1.0	0.6	0.4	0.8
Senior officer (O4-O10)	0.8	0.6	1.1	0.6	0.4	0.8
Warrant officer (W01-W05)	0.8	0.4	1.3	0.8	0.4	1.4
Occupation						
Combat-specific	1.1	0.7	1.9	0.9	0.5	1.6
Armor/motor transport	1.1	0.7	1.7	0.8	0.6	1.3
Pilot/air crew	1.6	0.9	2.8	1.2	0.7	2.5
Repair/engineering	1.1	0.9	1.3	1.1	0.9	1.3
Communications/intelligence	Reference	–	–	Reference	–	–
Health care	1.0	0.9	1.2	1.2	1.0	1.5
Other	1.1	0.9	1.3	1.0	0.8	1.2
Number of prior deployments						
0	Reference	–	–	Reference	–	–
1	0.9	0.8	1.1	0.9	0.7	1.1
2+	1.0	0.9	1.2	1.0	0.8	1.2
Prior risk factor diagnosis^a^						
Yes	2.3	1.9	2.7	2.0	1.7	2.3
No	Reference	–	–	Reference	–	–
Prior depressive or anxiety disorder diagnosis						
Yes	1.9	1.7	2.2	1.7	1.5	2.0
No	Reference	–	–	Reference	–	–
Prior PTSD diagnosis						
Yes	1.1	0.9	1.4	1.4	1.1	1.8
No	Reference	–	–	Reference	–	–

Abbreviations: aIRR, adjusted incidence rate ratio; LL, lower limit; UL, upper limit; y, years; PTSD, post-traumatic stress disorder.

^a^Includes prior diagnosis of hyperlipidemia, hypertension, diabetes or abnormal glucose, obesity, tobacco or nicotine dependence, or sleep apnea.

**Figure 2:**
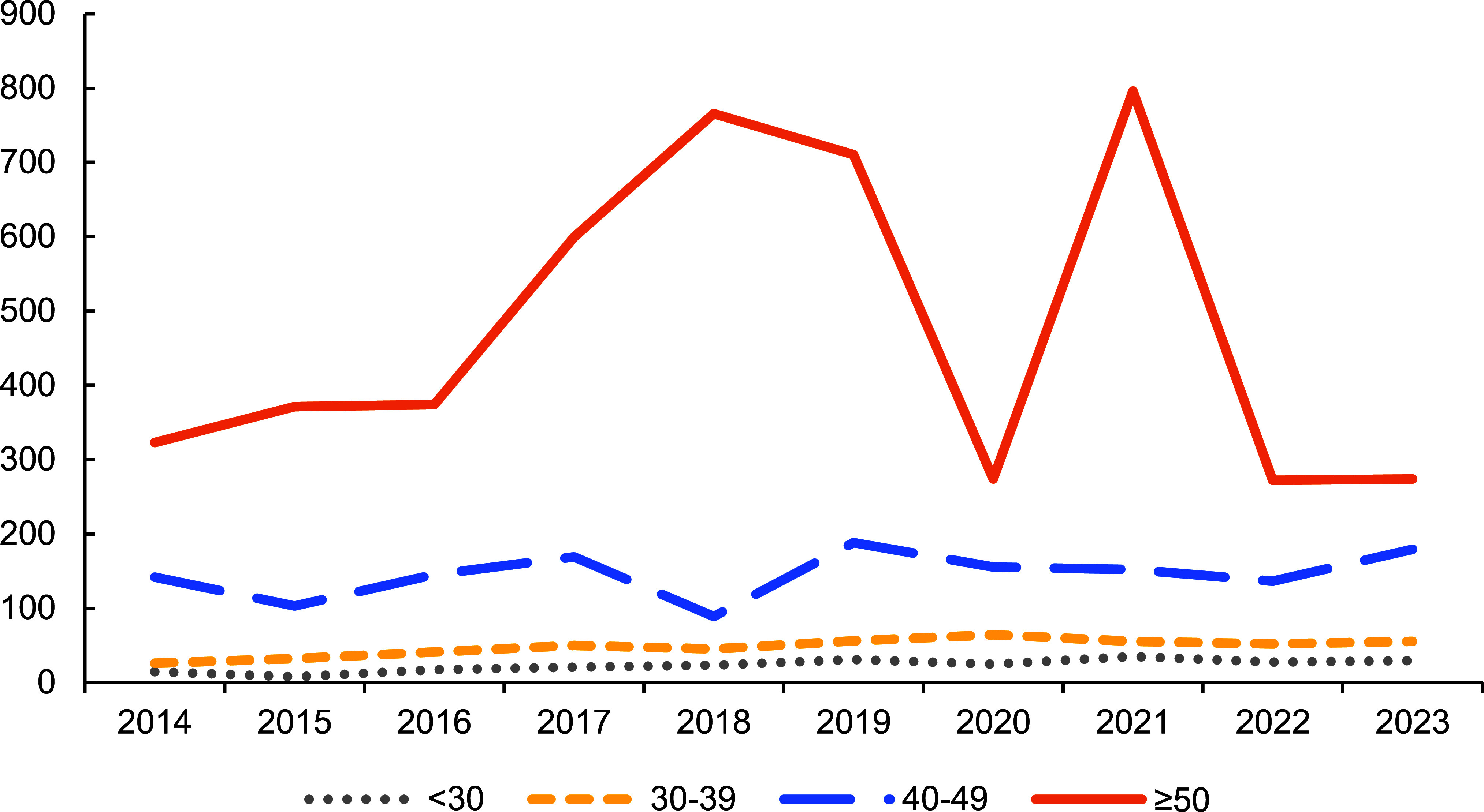
Annual Rates of Ischemic Heart Disease, by Age Group

**Figure 3:**
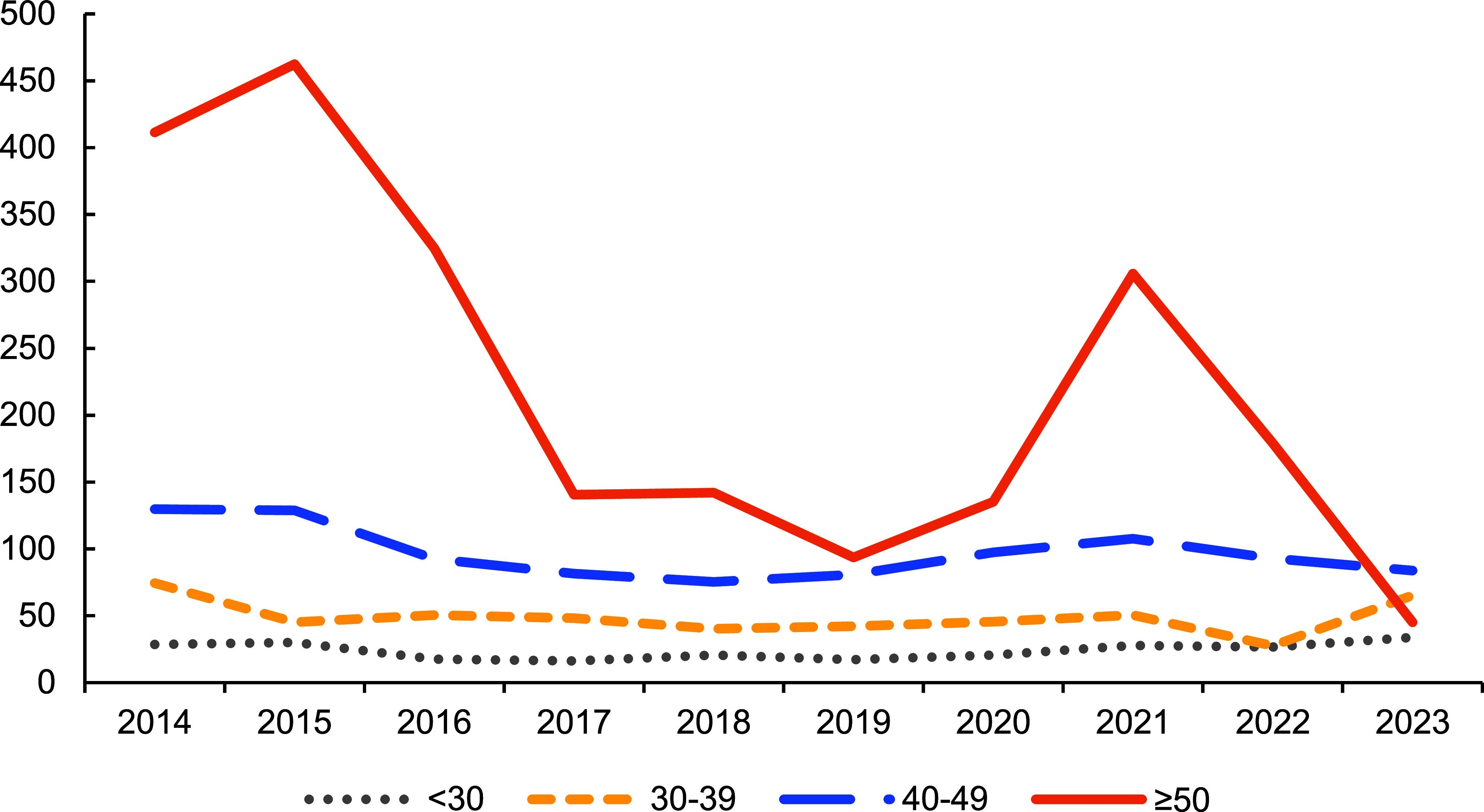
Annual Rates of Cerebrovascular Disease, by Age Group

## AUTHOR AFFILIATIONS

Uniformed University of the Health Sciences, Bethesda, MD: Maj Donici; Epidemiology and Analysis Branch, Armed Forces Health Surveillance Division, Defense Health Agency, Silver Spring, MD: Dr. Stahlman, Dr. Fan; Armed Forces Health Surveillance Division, Defense Health Agency, U.S. Department of Defense: CAPT Langton
